# Interleukin 33 exacerbates antigen driven airway hyperresponsiveness, inflammation and remodeling in a mouse model of asthma

**DOI:** 10.1038/s41598-017-03674-0

**Published:** 2017-06-26

**Authors:** L. C. Sjöberg, A. Zoltowska Nilsson, Y. Lei, J. A. Gregory, M. Adner, G. P. Nilsson

**Affiliations:** 1 Unit of Experimental Asthma and Allergy Research, Institute of Environmental Medicine, Karolinska University Hospital, Stockholm, Sweden; 2 Immunology and Allergy Unit, Department of Medicine, Karolinska University Hospital, Stockholm, Sweden; 3 Centre for Allergy Research, Karolinska Institutet, Karolinska University Hospital, Stockholm, Sweden

## Abstract

Interleukin 33 (IL-33) represents a potential link between the airway epithelium and induction of Th2-type inflammatory responses associated with the development of asthma. This study investigated the potential of IL-33 to exacerbate antigen driven asthma responses. An ovalbumin (OVA) asthma model was used in which sensitized C57BL/6 mice were exposed to IL-33 before each OVA challenge. IL-33 given to sensitized mice acted synergistically with antigen and aggravated airway inflammation, hyperresponsiveness and remodeling compared with mice that were only OVA sensitized and challenged and mice that were only exposed to IL-33. Elevated levels of local and systemic mast cell protease mMCP-1, as well as antigen-specific IgE production, were observed following IL-33 administration to sensitized mice. Similarly, exposing OVA-sensitized mice to IL-33 increased the Th2 cytokine levels, including IL-4, IL-5 and IL-13. Furthermore, IL-33 and OVA administration to OVA-sensitized mice increased ILC2s in the lung, suggesting a role for ILC2s in IL-33-mediated exacerbation of OVA-induced airway responses. Collectively, these findings show that IL-33 aggravates important features of antigen-driven asthma, which may have implications for asthma exacerbations.

## Introduction

Asthma is a chronic inflammatory airway disease currently afflicting more than 300 million people worldwide. It is characterized by airway hyperresponsiveness (AHR), inflammation and remodeling, that are associated with reversible airflow obstructions. Allergic asthma is the major phenotype of asthma^1^. It is characterized by a Th2-type inflammation, allergen-specific IgE induction and mast cell involvement. The immune system of atopic individuals that suffer from allergy responds abberantly to otherwise harmless substances such as pollen. However, only a fraction of individuals express their atopy in the form of asthma, indicating that additional factors are driving the reaction. As recent evidence suggest that asthma is a disorder of the airway epithelium which perpetuates chronic airway inflammation by deffective responses to environmental agents such as viral and allergen exposure^2^, epithelial responses may be the critical link for turning atopy into asthma.

Interleukin 33 (IL-33) represents one of the potential signals from the epithelial cells that trigger the development of asthma. IL-33 has been shown to be expressed in structural cells such as epithelial and smooth muscle cells and its levels have been correlated to asthma severity^3, 4^. Furthermore, polymorphisms in the genes for IL-33, its receptor ST2, and the downstream signaling have been associated with asthma development and severity in genome wide association studies^5–7^. It has been demonstrated that IL-33 is released from epithelial cells^8^, both during necrosis^9^ and by exposure to allergens such as ovalbumin (OVA)^10^, house dust mite^11^ and *Alternaria* ^12^. Smooth muscle cells have also demonstrated a steroid resistant release of IL-33 in response to inflammatory stimuli^3^. Using mice deficient in the IL-33 receptor or treated with either the soluble IL-33 receptor or with anti-IL-33, different features of asthma have been shown to be reduced, including the time to resolution of the allergic inflammation^10, 13–17^.

IL-33 signals via ST2, also known as IL1RL1, along with the co-receptor IL-1RAcP^18^. Several cell types of both the innate and adaptive immune system express the ST2 receptor and thus have the ability to respond to IL-33, including mast cells, type 2 innate lymphoid cells (ILC2s), eosinophils, and macrophages^19^.

Previous studies have demonstrated that intranasal IL-33 administration leads to eosinophil infiltration, promotes remodeling of the airways, and induces AHR^13, 20^. Recently we showed that IL-33 exacerbates the early allergic reaction through a mechanism involving increased synthesis, storage and secretion of the mast cell mediator serotonin^21^. In the current study we have taken a new approach where we define the role of IL-33 in exacerbating antigen-induced asthma. To investigate this we have studied airway inflammation, remodeling and AHR in a mouse model of asthma. We demonstrate that IL-33 acts synergistically with antigen leading to exacerbations of airway responses.

## Results

### IL-33 acts in synergy with antigen to induce robust airway inflammation and airway hyperresponsiveness

**Figure 1:**
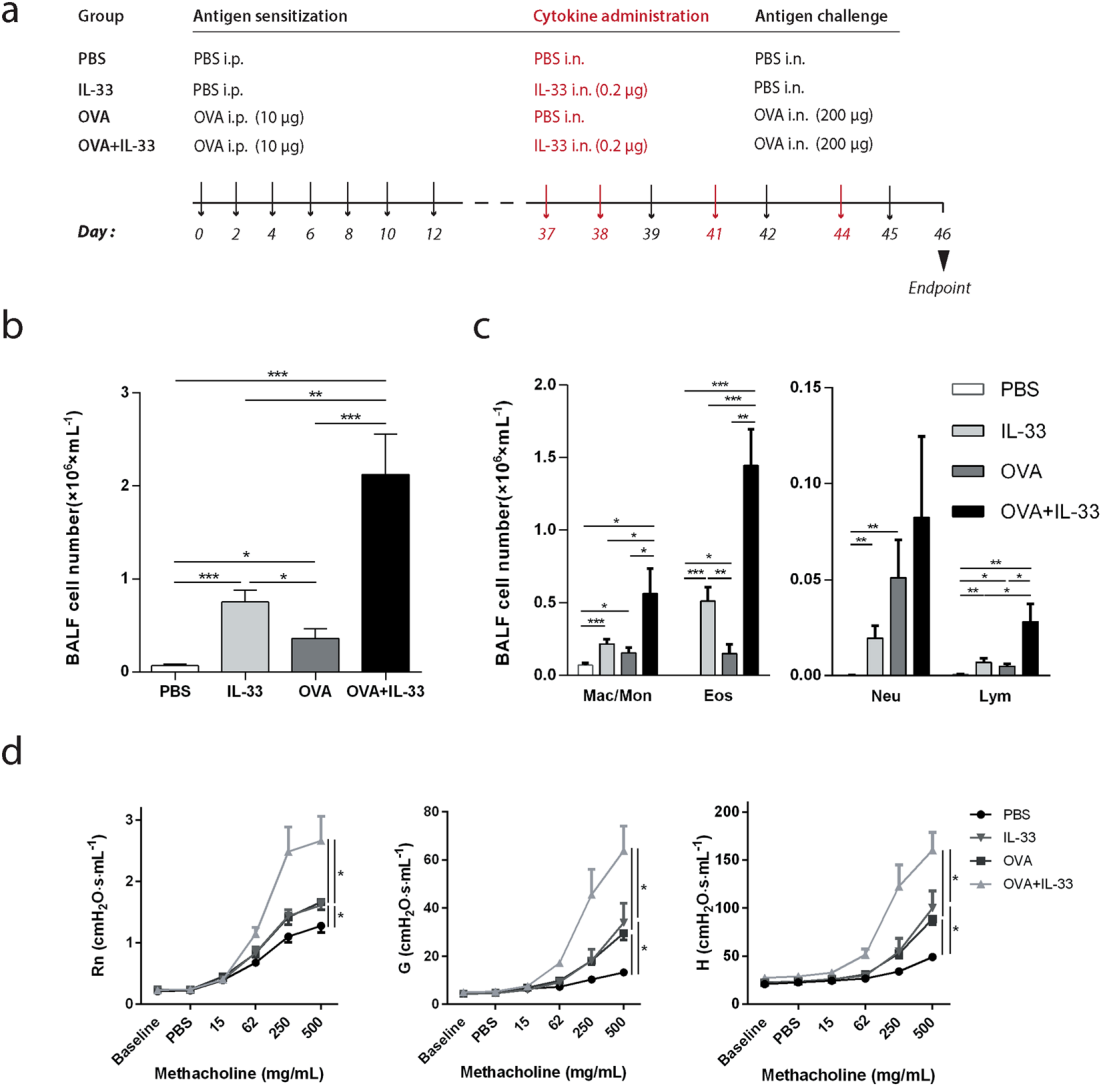
IL-33 potentiates antigen induced inflammation in BALF and airway hyperresponsiveness. (**a**) Schematic representation of the exposure protocol. Sham-or OVA-sensitized C57BL/6 mice received intranasal instillations of IL-33 or PBS. (**b**) Total inflammatory cells in BALF were counted. (**c**) Differential cell counts in BALF were determined. Results expressed as cells/mL for macrophages/monocytes, eosinophils, neutrophils and lymphocytes. (**d**) Airway hyperresponsiveness (AHR) in response to inhaled methacholine was measured in sham- or OVA-sensitized C57BL/6 mice that received intranasal instillations of IL-33 or PBS. Maximal responses to increasing doses of methacholine are shown for newtonian resistance (*Rn*), tissue damping (*G*) and tissue elastance (*H*) (**b**–**d**) *p < 0.05, **p < 0.01, ***p < 0.001 (ANOVA, Bonferroni). Results are pooled data from four independent experiments (mean ± SEM of n = 9–10 mice for each group).

To investigate the influence of IL-33 in antigen-induced airway pathology we modified a previously established mast cell-dependent asthma model, which is based on sensitization with repetitive i.p. injections of low-dose OVA without adjuvant followed by intranasal OVA challenge^22^. Preceding each OVA provocation we introduced intranasal instillations of IL-33 administrated to C57BL/6 mice (Fig. 1a). Bronchoalveolar lavage fluid (BALF), serum and lung specimens were collected 24 h after the last antigen challenge. Exposure to OVA alone or IL-33 alone both induced a marked increase in the total number of inflammatory cells in BALF, although exposure to IL-33 resulted in a more pronounced response than to OVA alone (Fig. 1b). Importantly, IL-33 exposure in OVA-sensitized mice further increased the number of inflammatory cells in BALF in a synergistic manner. The observed airway inflammation was predominantly reflected in a marked increase in BALF eosinophils followed by macrophages, neutrophils and lymphocytes (Fig. 1c).

Since asthma is characterized by airway hyperresponsiveness (AHR) and altered levels of IL-33 have been associated with asthma, we went further to investigate the effect of IL-33 on AHR in the OVA-induced mouse asthma model. AHR was assessed in anesthetized and tracheotomized mice. Resistances of the conducting airways (Newtonian resistance (Rn)) as well as peripheral lung function (tissue damping (G) and tissue elastance (H)), in response to aerosolized methacholine, were determined. Both OVA sensitization and IL-33 administration alone led to increased AHR in all parameters measured (Fig. 1d). Notably, an enhanced response was observed in all three lung parameters when OVA sensitized mice were exposed to IL-33.

### IL-33 aggravates lung tissue inflammation and structural remodeling in OVA-sensitized mice

**Figure 2:**
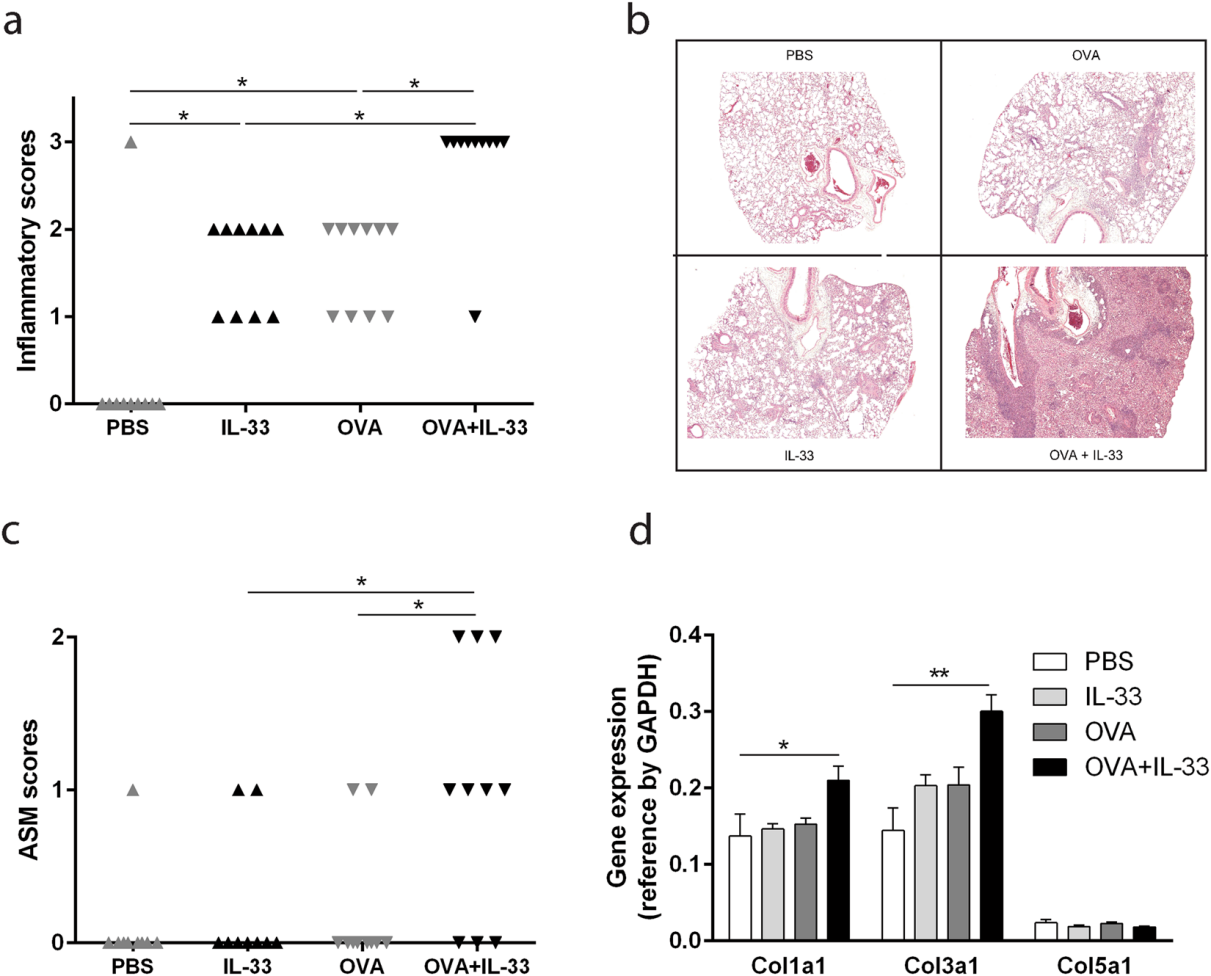
Lung tissue inflammation and structural airway remodeling is enhanced by IL-33 administration in OVA-sensitized mice. (**a**) Scoring of inflammation in lung tissue. 0 = no inflammatory cell infiltration, 1 = inflammatory cells present in central airways, 2 = inflammatory cells present in central airways and parenchyma, 3 = massive infiltration of inflammatory cell in the central airways as well as in the parenchyma. (**b**) Representative images of haematoxylin and eosin staining of lung tissue graded in panel 2a. (**c**) Scoring of the ASM cell layer around the central airways. The thickness of the ASM cell layer was assessed and a relative score was assigned to each sample, where 0 = normal, 1 = thickened and 2 = substantially thickened ASM cell layer. ASM = airway smooth muscle. (**a**,**c**) *p < 0.05 (Chi-square test for trend). Results are pooled data from four independent experiments (mean ± SEM of n = 9–10 mice for each group). (**d**) Relative quantitation (RQ) of mRNA levels of *Col1a1*, *Col3a1* and *Col5a1* were assessed in lung specimens. *p < 0.05, **p < 0.01 (ANOVA, Bonferroni). Results are pooled data from four independent experiments (mean ± SEM of n = 3–4 mice in each group).

Further investigation into the effects of IL-33 on inflammation in the lung was performed by haematoxylin and eosin staining of lung sections. The inflammation was intensely increased in lung tissue from OVA-sensitized mice that received intranasal instillations of IL-33 compared to the groups that received only OVA or IL-33 (Fig. 2a,b).

To elucidate whether IL-33 affected tissue remodeling, we analyzed the thickness of the smooth muscle layer circumscribing the central airway and the expression of three collagen genes in lung tissue. OVA-sensitization or IL-33 administration alone did not increase the thickness of the airway smooth muscle layer compared to sham-sensitized mice that received intranasal PBS (Fig. 2c). However, in a fashion similar to other parameters measured, IL-33 administration to OVA-sensitized mice prominently enhanced the thickness of the smooth muscle layer. Expression levels of both *Col1a1* and *Col3a1*were significantly induced in the combination group of OVA and IL-33 compared to the PBS control group (Fig. 2d). There were no significant differences between groups in *Col5a1* expression levels (Fig. 2d).

### IL-33 induces mast cell activation but not increased mast cell numbers

**Figure 3:**
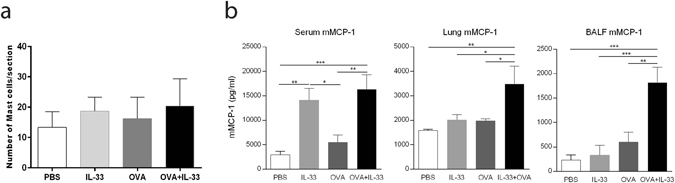
IL-33 treatment of OVA-sensitized mice leads to enhanced mast cell activation but no change in mast cell number. (**a**) Number of mast cells was determined in lung sections, counted in nine sections per mouse. (**b**) Mast cell protease mMCP-1 levels were measured in BALF, lung and serum. *p < 0.05, **p < 0.01, ***p < 0.001 (ANOVA, Bonferroni). Results are pooled data of four independent experiments (mean ± SEM for (**a**) n = 7–9 and (**b**) n = 4–10 mice in each group).

Since IL-33 has several prominent effects on mast cell functions^23^, we explored if IL-33 exposure in sensitized mice would lead to mast cell expansion in the lung. IL-33 treatment showed a tendency, though not statistically significant, to increase the number of mast cells, both in sham sensitized mice as well as in OVA sensitized mice (Fig. 3a). We then progressed to investigate whether mast cells were activated by IL-33 exposure by measuring the levels of mast cell protease mMCP-1 in serum, lung homogenates and BALF, reflecting systemically released, locally stored and released mMCP-1, respectively. On a systemic level, as estimated by serum mMCP-1, IL-33 alone gave rise to a similar response as when IL-33 was given to sensitized animals, while OVA alone did not differ significantly from the PBS group (Fig. 3b). Local lung production and release of mMCP-1 in BALF was enhanced only in sensitized mice that were instilled with IL-33.

### IL-33 in combination with antigen exposure leads to a prominent increase in OVA specific IgE and lung cytokines

**Figure 4:**
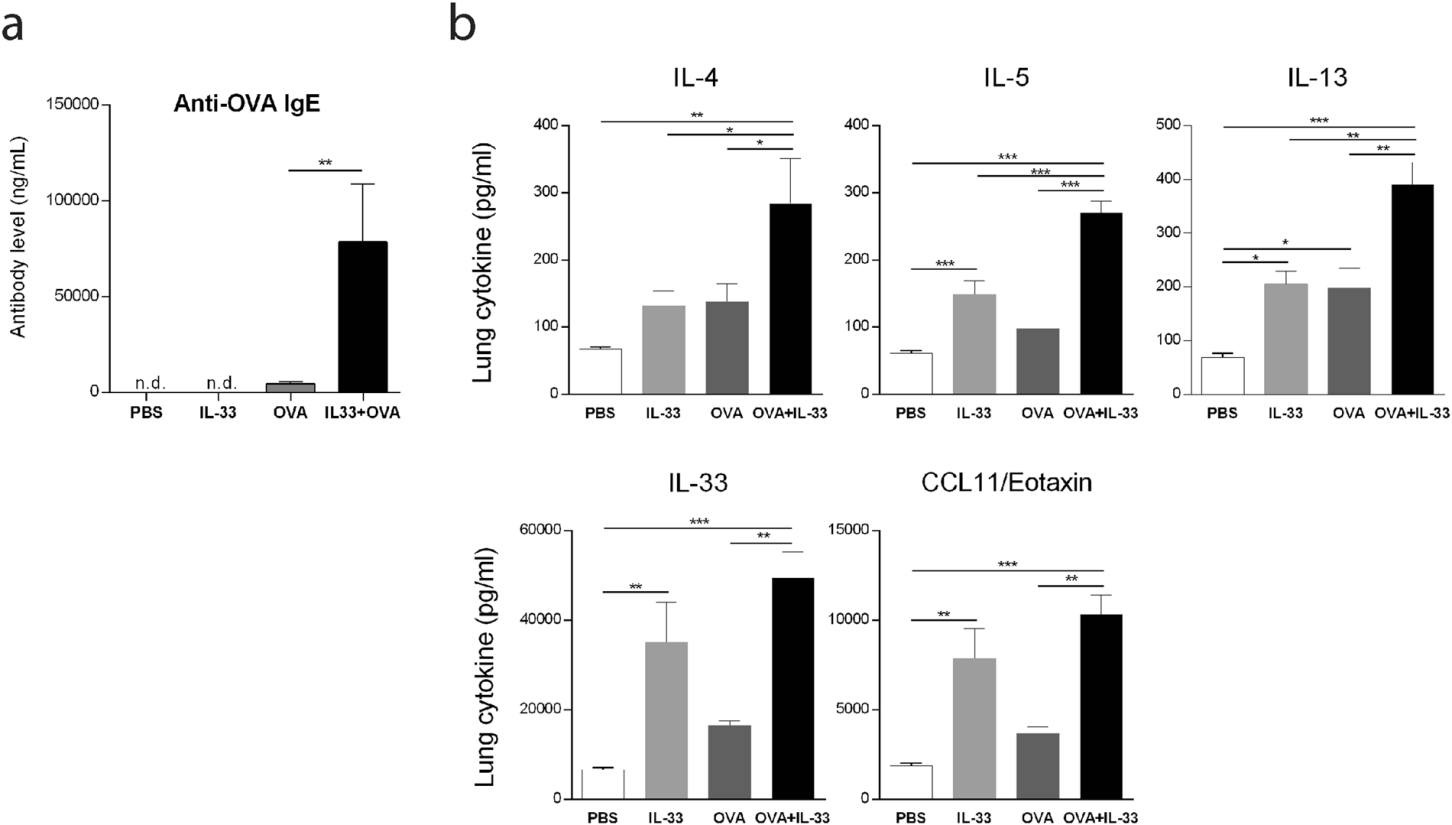
IL-33 exposure enhances antigen induced OVA specific IgE and increases lung cytokine levels in OVA-sensitized mice. (**a**) Serum specimens were analyzed to determine the levels of anti-OVA IgE Abs. n.d. = not detectable. **p < 0.01 (ANOVA, Bonferroni). Data are representative of two independent experiments with similar results (mean ± SEM of n = 5–6 mice in each group) (**b**) Lung homogenates were analyzed for cytokine levels. *p < 0.05, **p < 0.01, ***p < 0.001 (ANOVA, Bonferroni). Results are pooled data of four independent experiments (mean ± SEM of n = 4–6 mice in each group).

IL-33 has previously been shown to increase total IgE levels in mice^18, 24^. We therefore investigated the role of IL-33 on antigen-specific IgE production in the OVA exposure model. The production of OVA specific serum IgE was enhanced profoundly by exposing OVA-sensitized C57BL/6 mice to IL-33 (Fig. 4a). IL-33 exposure alone led to an increase in IL-5, IL-13, IL-33 and CCL11 (eotaxin) levels. OVA alone increased IL-13 levels in the lungs compared to PBS control, but did not significantly alter the levels of any other of the cytokines measured (Fig. 4b). When OVA-sensitized mice were exposed to IL-33, all cytokines were elevated compared to the PBS group, and IL-4, IL-5 and IL-13 were increased compared to the IL-33 or OVA group. Notably, IL-4 was only increased when IL-33 was given to OVA-sensitized mice, but not in the IL-33 and OVA groups, compared to the PBS control group.

### IL-33 enhances the accumulation of pulmonary ILC2s in OVA-sensitized mice

**Figure 5:**
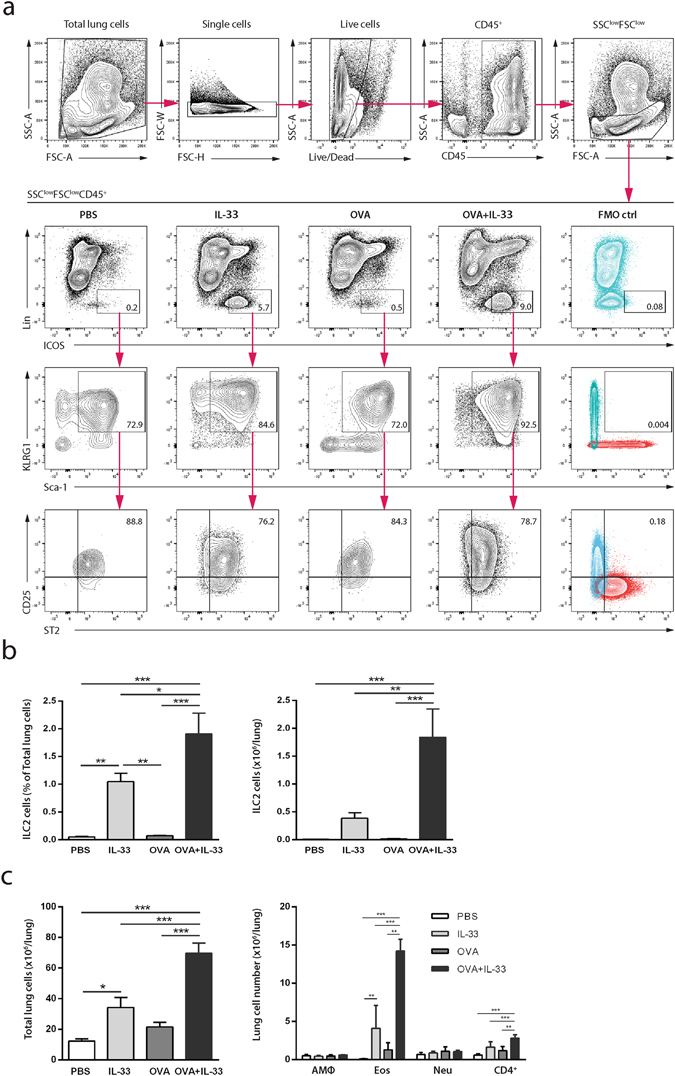
IL-33 enhances the accumulation of pulmonary ILC2s in OVA-sensitized mice. Flow cytometric analysis of lung cells. (**a**) ILC2s were identified as SSC^low^FSC^low^CD45^+^Lin^−^ICOS^+^KLRG1^+^Sca-1^+^CD25^+^ ST2^+^ cells. Gates were set based on FMO controls, FMO ctrl = overlay of the two FMO controls for each respective plot. The representative FMO plots and plots showing the representative SSC^low^FSC^low^CD45^+^ gating belong to the OVA + IL-33 group. (**b**) Frequency and numbers of pulmonary ILC2s. (**c**) Quantification of inflammatory lung cells. Alveolar macrophages were identified as CD45^+^ SiglecF^+^ CD11c^+^, eosinophils as CD45^+^SiglecF^+^CD11c^−^CD11b^+^Gr-1^lo^, neutrophils as CD45^+^ SiglecF^−^ CD11c^−^Gr-1^hi^CD11b^hi^ and CD4+ cells as CD45^+^CD11b^−^ CD4^+^ (frequency and representative plots including FMO controls can be found in Supplementary Fig. S1). *p < 0.05, **p < 0.01, ***p < 0.001 (ANOVA, Bonferroni). Results are pooled data of four independent experiments (mean ± SEM of n = 8 mice in each group).

Several cell types of the immune system including mast cells, macrophages, Th2 cells, eosinophils, neutrophils and ILC2s have the potential to respond to IL-33^19^. Particularly, ILC2s have been identified as early responders to IL-33 and a potent source of Th2 cytokines including IL-5 and IL-13^25^. Accordingly, we chose to investigate how ILC2s as well as macrophages, eosinophils, neutrophils and CD4^+^ cells, were affected in our model. ILC2s were identified as SSC^low^FSC^low^Lin^−^CD45^+^ICOS^+^KLRG1^+^Sca-1^+^CD25^+^ST2^+^ cells (Fig. 5a). The majority of cells in the SSC^low^FSC^low^Lin^−^CD45^+^ICOS^+^ gate expressed both KLRG1 and Sca-1. Likewise, most cells in the SSC^low^FSC^low^Lin^−^CD45^+^ICOS^+^KLRG1^+^Sca-1^+^ gate expressed both CD25 and ST2. ILC2s in the lung increased following IL-33 exposure, while ILC2s in only OVA-sensitized mice were not significantly affected (Fig. 5b). Interestingly, there was a prominent increase in the accumulation of ILC2s in the lung when OVA-sensitized mice were exposed to IL-33. The total lung cell numbers were increased following IL-33 exposure of naïve mice and increased further when IL-33 was given to OVA-sensitized mice (Fig. 5c). Eosinophils (CD45^+^ SiglecF^+^ CD11c^−^ CD11b^+^Gr-1^low^) were the most abundant lung cell type investigated and were induced after IL-33 instillation alone and considerably augmented in combination with OVA-sensitization (Fig. 5c, Supplementary Fig. S1). An increase in CD4^+^ cells (CD45^+^CD11b^−^CD4^+^) was seen in IL-33 exposed OVA-sensitized mice. However, alveolar macrophages (CD45^+^SiglecF^+^CD11c^+^) and neutrophils (CD45^+^ SiglecF^−^ CD11c^−^CD11b^hi^Gr-1^hi^) did not increase significantly in any treatment group.

## Discussion

Recently, we showed that IL-33 exacerbates the early allergic bronchoconstriction through a mechanism involving increased synthesis, storage and secretion of the mast cell mediator serotonin^21^. In the present study, we provide evidence that IL-33 exacerbates antigen-induced asthmatic responses, including airway hyperresponsiveness, inflammation and remodeling, mast cell activation, production of IgE and cytokines including IL-4, IL-5 and IL-13, and accumulation of pulmonary ILC2s. We propose a central role for mast cells and ILC2s in these IL-33 mediated processes.

IL-33 administered to sham-sensitized mice as well as OVA-sensitization and challenge, caused an increase in macrophages/monocytes, eosinophils, neutrophils and lymphocytes in BALF. The increase in the total number of inflammatory cells we observed in BALF following IL-33 administration to naïve mice is consistent with previous findings^20^. Importantly, the number of inflammatory cells in BALF was significantly enhanced by the introduction of IL-33 to OVA-sensitized mice. Following OVA sensitization and IL-33 administration, eosinophils were the cell type that preferentially recruited to the BALF. Intranasal instillations of IL-33 have previously been shown to induce an increase of eosinophils in murine airways and the OVA protocol, which the protocol used herein is based on, has been shown to induce a mixed inflammatory response^20, 26^.

IL-33 administration to OVA-sensitized and challenged mice led to a synergistic increase in AHR both centrally and peripherally. Alone, IL-33 induced marked AHR that was comparable to the OVA-induced AHR in our model. This is consistent with other studies in which intranasal IL-33 administration produced AHR in BALB/c mice^20, 27^. This phenomenon was also observed in RAG-2 (−/−) mice, lacking T and B cells, suggesting that IL-33 induced development of AHR occurs independent of the adaptive immune system^20^.

We could also demonstrate how IL-33 together with OVA-sensitization and challenge affects lung tissue inflammation and remodeling. Separately, IL-33 or OVA administration induced lung inflammation. IL-33 administration combined with OVA further increased the level of lung inflammation, which was prominent both in large airways as well as in the peripheral lung tissue. To assess potential changes in airway remodeling, we chose to measure the expression levels of the collagen genes *Col1a1*, *Col3a*1 and *Col5a1* because they have been shown to be involved in reticular basement membrane fibrosis in asthma^28^. Expression levels of *Col1a1* and *Col3a*1 were increased in mice that received OVA in combination with IL-33. It has previously been shown that collagen levels in the lung are increased following two weeks of intranasal IL-33 instillations in BALB/c mice^13^. This was not observed in our study in which C57BL/6 mice were exposed to IL-33 for shorter duration. However, an increase in collagen expression could be observed when IL-33 was combined with OVA. Neither OVA nor IL-33 administration alone caused an increase in thickness of the smooth muscle layer around the central airways. However, an increase in smooth muscle thickness was observed when OVA-sensitized and challenged mice were exposed to IL-33. Increased collagen deposition and smooth muscle thickening around the airways reduces the elasticity of the airways and as such, these data indicate that the combined effects of OVA and IL-33 exposure on remodeling of the airways can lead to airflow obstructions.

This study is based on an OVA-model, which has previously been reported to be mast cell dependent^22^. For this reason, we thought to investigate how the introduction of intranasal instillation of IL-33 would affect mast cell activity. It has previously been shown that mast cell survival, maturation, adhesion and activation are increased by IL-33 *in vitro* ^29, 30^ and *in vivo* ^31^. Because, we could not observe an increase in mast cell density in the lungs following treatment in any of the experimental groups in our model, we hypothesized that the exacerbated airway hyperresponsiveness by IL-33 in OVA exposed mice could be explained by an enhancement of mast cell mediator release as opposed to an increase in mast cell numbers. Indeed, our data show increased levels of the mast cell mediator mMCP-1 indicating mast cell degranulation. Previously we demonstrated *in vitro* that IL-33 can act directly on sensitized mast cells to increase the storage of serotonin that is released following IgE-receptor cross-linking^21^. Furthermore, it has been reported that intraperitoneal IL-33 administration to naïve mice amplifies IgE synthesis, independent of antigen, via IL-4 released mainly by mast cells and eosinophils^24^. Here, we show that IL-33 can enhance antigen-specific IgE production in sensitized mice. We were also able to measure elevated levels of IL-4 in lung homogenate in the IL-33 and OVA exposed mice. Taken together, these data suggest that IL-33 has capacity to enhance mast cell responses via an indirect amplification of antigen-specific IgE driven by increased IL-4 levels as well as directly increasing mast cell mediator production and storage, cumulatively leading to more frequent and potent mast cell degranulation.

ILC2s have been identified as important players in allergic asthma and their accumulation can be induced via IL-33 in response to allergens such as *Alternaria* and house dust mite, as well as influenza virus^32–34^. Importantly, ILC2s have the capacity to produce critical amounts of IL-5 and IL-13 after intranasal challenge with several different allergens including OVA in murine models of allergic asthma^33^. ILC2s thus constitute a potential source of the elevated Th2 lung cytokines IL-5 and IL-13, which we observed following IL-33 administration in OVA sensitized and challenged mice. IL-5 is known to induce the development, recruitment and activation of eosinophils, whereas IL-13 promotes mucus hyperproduction, airway remodeling and subsequent AHR^13, 35–41^. The finding that IL-33 enhanced induction of IL-5 and IL-13 in OVA exposed mice could explain the increased accumultion of eosinophils in BALF and lung tissue, and the enhanced AHR. Interestingly, we observed a prominent increase in pulmonary ILC2s when OVA-sensitized mice were exposed to IL-33, suggesting that ILC2s could be involved in the IL-33 mediated exacerbations of allergen-induced asthmatic responses presented herein. Notably, OVA exposure alone did not induce accumulation of ILC2s in the lung, which could be explained by the non-significant increase in IL-33 lung levels, which we see in our model following OVA exposure. Similar results have been reported previously in a different mouse model of OVA-induced asthma, in which ILC2s were not accumulated in the lung^42^.

In this study we provide evidence that IL-33 exacerbates antigen-driven features of allergic asthma and we propose that mast cells and ILC2s have a central role in driving these processes. More functional studies are needed to establish whether the observations we report herein can be coupled to the exacerbation of asthma symptoms observed in sensitized individuals. Exacerbations of allergic asthma could be explained by an inappropriate and excessive release of IL-33 caused by epithelial triggers such as viral infection, which is the most common cause of asthma exacerbations, as well as allergen exposure^5, 43, 44^. Collectively, our data suggests that IL-33 constitutes a potential target for the management and prevention of asthma exacerbations^43, 45^.

## Materials and Methods

### Mice

Male C57BL/6 mice (6–8 weeks; Charles River Laboratories, Sulzfeld, Germany) were housed with a 12 hour light/dark cycle and were given food and water ad libitum. All animal handling and experimentation was conducted in accordance with ethical permits approved by the Regional Committee of Animal Experimentation Ethics (Stockholm, Sweden).

### *In vivo* allergic sensitization, challenge and administration of IL-33

Mice were sensitized and challenged using a previously described protocol known to induce mast cell-dependent allergic inflammation^22^. To study the effects of IL-33, the protocol was modified by introducing intranasal IL-33 instillations to sensitized mice before each antigen challenge. Mice were sensitized via i.p. injections with 10 µg/dose OVA (grade V; Sigma-Aldrich) in 100 µL saline or sham-sensitized with the equivalent volume of saline alone on days 0, 2, 4, 6, 8, 10 and 12 (Fig. 1a). The mice were then challenged with 200 µg/dose OVA in 20 µL PBS or PBS alone via i.n. instillation on days 39, 42 and 45. IL-33 (eBioscience, San Diego, CA, USA) (0.2 µg of IL-33 in 20 µL PBS) or PBS alone were given, before each OVA-challenge, via i.n. instillation on days 37, 38, 41 and 44. Lung mechanics measurements and tissue collection were performed 24 hours following the final OVA challenge.

### Collection and preparation of bronchoalveolar lavage, serum and lung specimens

The lungs of each mouse were lavaged three times with 800 µL of cold PBS and the recovered volume was collected as bronchoalveolar lavage fluid (BALF). The cell pellet was separated from the supernatant and counted. The supernatant was saved in −20 °C for later analysis. Blood was collected by cardiac puncture with a 20-gauge needle. Serum was prepared via centrifugation in serum tubes (Sarstedt, Helsingborg, Sweden) and stored at −20 °C until further analyses. The left lung lobes from each mouse were removed and fixed in 4% formaldehyde (Histolab, Stockholm, Sweden). Right lung lobes were snap-frozen and stored at −80 °C. For flow cytometric analysis, using different set of mice, lungs were perfused with cold PBS through the right ventricle and left and right lobes were collected for preparation of single-cell suspensions.

### Inflammatory cell counts in bronchoalveolar lavage fluid

The total number of inflammatory cells present in the BALF was determined via hemocytometer (FastRead102, Immune Systems LTD, United Kingdom) following staining with Turk’s solution (Histolab Products AB, Gothenburg, Sweden). 50,000 cells per sample were plated on glass slides by cytospin centrifugation. To visualize inflammatory cells, the slides were stained with May-Grünwald and Giemsa stain (Sigma-Aldrich). Differential cell counts were performed by counting 300 cells from BALF per mouse.

### *In vivo* measurement of pulmonary mechanics

Mice were anesthetized by subcutaneous injection with a combination of hypnorm (2.5 µL/g; VetaPharma Ltd, Leeds, UK) and midazolam (12.5 µg/g; Hameln Pharmaceuticals gmbh, Hameln, Germany). Anesthetized mice were placed on a heating pad (37 °C) and tracheotomized with a blunted 18-gauge needle. Mice were then connected to the flexiVent animal ventilator (Scireq, Montreal, Canada). Ventilation was performed at 2.5 Hz in a quasi-sinusoidal fashion in order to generate a sinusoidal pressure waveform during lung inflation. The tidal volume was set at 12  ml.kg^−1^ body weight with a positive end-expiratory pressure (PEEP) of 3 cm H_2_O. Once ventilation was initiated, a bilateral thoracotomy was performed in order to equalize pleural pressure and to eliminate the possibility of spontaneous chest wall movements interfering with lung mechanics measurements. In order to induce airway hyperresponsiveness, increasing doses (0, 15.6, 62.5, 250 and 500 mg/ml) of aerosolized acetyl-β-metacholine (Sigma-Aldrich) were delivered directly to the lung via an Aeroneb nebulizer (Scireq). Respiratory impedance was measured using the forced oscillation technique and was fit with the constant phase model where Newtonian resistance *Rn* reflecting the resistance of the conducting airways, *G* characterizing tissue dampening and *H* characterizing tissue stiffness or tissue elastance were determined^46^.

### Investigation of mast cell numbers and inflammation in lung tissue

The left lung lobes were dehydrated, embedded in paraffin and cut into 5 µm sections and placed on glass slides. The sections were then deparaffinized and stained with 1% toluidine blue (Sigma-Aldrich) followed by counterstain with fast green (Polysciences, Inc., Warrington, PA, USA) to detect mast cells. Mast cells were counted in nine sections per mouse, taken from three cross-sectional places distal to the hilum, each separated by at least 20 µm. To evaluate inflammatory cell infiltration and smooth muscle layer thickness, sections were stained with haematoxylin and eosin (HistoLab). Inflammatory cell infiltration was scored using the following scale: 0 = no inflammatory cell infiltration, 1 = inflammatory cells present centrally, 2 = infiltration centrally and some spread to the parenchyma and 3 = massive infiltration centrally as well as in the parenchyma. The thickness of the smooth muscle cell layer around the central airway was measured and each sample was then given a relative score; 0 = low, 1 = intermediate, and 2 = high ASM score based on the thickness of the ASM layer.

### Quantification of collagen gene expression

RNA was extracted and purified from the superior and middle right lung lobes using TRIzol Reagent (Life Technologies Corporation, Carlsbad, CA, USA) and RNeasy Mini Kit (QIAGEN GmbH, Hilden, Germany) according to the manufacturer’s instructions. cDNA was synthesized using iScript cDNA Synthesis Kit (Bio-Rad Laboratories, CA, US) according to the manufacturer’s instructions. Real-time quantitative PCR was performed using Power SYBR® Green PCR Master Mix (Applied Biosystems, Foster City, CA, USA) and SYBR® Green primers (Sigma-Aldrich) for *collagen, type I, alpha 1* (*Col1a1*) (forward: ACGCATGGCCAAGAAGACA, reverse: TACAGATCAAGCATACCTCGGGTT), *collagen, type III, alpha 1* (*Col3a1*) (forward: CTAGACTGCCCCAACCCAGA, reverse: AGGTCCATGGCCATCAGGA) and *collagen, type V, alpha 1* (*Col5a1*) (forward: GCCCGGGCCTGAAGAGTA, reverse: CCACTTGCCATCGGACAAG). Primers for the housekeeping genes *glyceraldehyde-3-phosphate dehydrogenase* (*Gapdh*) (forward: CATGGCCTTCCGTGTTCCTA, reverse: TGCTTCACCACCTTCTTGATG) and *beta*-*actin* (*Actb*) (forward: TGGGTCAGAAGGACTCCTATGTG, reverse: CGTCCCAGTTGGTAACAATGC) were also included. Quantification was performed on Bio-rad CFX96 Real-Time PCR Detection System and relative quantitation (RQ) was calculated using the comparative C_T_ (2^−ΔΔCt^) method. Expression of the gene of interest was then normalized to *Gapdh*.

### ELISA

The levels of OVA-specific IgE in serum were measured using an ELISA kit following the procedure recommended by the manufacturer (MD Bioproducts, Zurich, Switzerland). mMCP-1 levels were determined in BALF and serum using aELISA kit (eBioscience, San Diego, CA). Lung protein was extracted from snap-frozen samples of pulmonary tissue with a cell lysis kit (Bio-plexTM Cell Lysis kit, Bio-Rad) supplemented with proteinase inhibitors (Sigma-Aldrich). The cytokines IL-4, IL-5, IL-13, IL-33 and mast cell protease mMCP-1 were quantified in the supernatants of lung homogenates by ELISA kits according to the manufacturer’s instructions (eBioscience, San Diego, CA). CCL11 levels in lung homogenates were determined using an ELISA kit (R&D Systems, Minneapolis, MN).

### Surface staining and flow cytometric analysis

Single-cell suspensions of lung tissue were prepared from right and left lung lobes as described^47^ with some modifications. Briefly, lung lobes were cut into small segments and digested in RPMI medium containing Liberase TM, research grade (50 ug/ml) and DNase I (50 ug/ml), (Roche, Mannheim, Germany). After digestion, lung tissue was homogenized manually over a 100-μm nylon strainer using the flat end of a plunger. Cells were surface stained with fluorescently labeled antibodies to CD45, CD3, CD4, CD19, CD5, CD8a, TCRβ, CD49b, NK1.1, CD11b, CD11c, FcεRIα, TER119, F4/80, ICOS, KLRG1, Sca-1, CD25, Siglec-F, GR-1 and ST2 as described in the antibodies section. Appropriate isotype controls were used to verify proper Fc-blocking and exclude the possibility of unspecific binding. FMO controls were used to set the gates and dead cells were excluded using Live/dead fixable blue dead cell stain kit (Invitrogen, Carlsbad, U.S.). Samples were analyzed using LSRFortessa cytometer (BD Biosciences, Franklin Lakes, NJ, USA). Flow cytometric data were analyzed using the FlowJo software (Tree Star Inc., Ashland, OR, USA). ILC2 cells were characterized as CD45^+^Lineage^−^ICOS^+^KLRG1^+^Sca-1^+^CD25^+^ ST2^+^ (Fig. 5a). Markers in the lineage cocktail consisted of CD3, CD4, CD19, CD5, CD8a, TCRβ, CD49b, NK1.1, CD11b, CD11c, Gr-1, FcεRIα, TER119 and F4/80. Macrophages were identified as CD45^+^ SiglecF^+^CD11c^+^, eosinophils as CD45^+^SiglecF^+^CD11c^−^CD11b^+^Gr-1^lo^, neutrophils as CD45 + SiglecF^−^ CD11c^−^CD11b^hi^Gr-1^hi^ and CD4^+^ cells as CD45^+^ CD11b^−^ CD4^+^ (Supplementary Fig. S1) as previously described^48^.

### Antibodies

Cells were pre-treated with anti-CD16/32 (clone 2.4G2, BD Pharmingen) before staining to prevent unspecific Fc-binding of antibodies. ILC2 cells were identified by the following antibodies: PE-conjugated anti-lineage antibodies CD3 (clone 17A2), CD4 (clone RM4-4), CD8a (clone 53–6.7), CD11b (clone M1/70), CD19 (clone 6D5), CD5 (clone 53–7.3), TCRβ (clone H57-597), FcεRIα (MAR-1), TER119 (TER-119), CD49b (clone DX5), NK1.1 (PK136), F4/80 (T45-2342), Gr-1 (RB6-8C5), CD11b (M1/70) and CD11c (N418); APC-Cy7 conjugated CD45 (clone 30-F11); ICOS conjugated to Alexa Fluor 647 (clone C398.4 A), BV421-conjugated KLRG1 (2FI), PeCy7 conjugated Sca-1 (D7), PerCPCy5.5-conjugated CD25 (PC61), and FITC-conjugated T1/ST2 (clone DJ8). Macrophages, eosinophils, neutrophils and CD4^+^ cells were characterized using APC-Cy7 conjugated CD45 (clone 30-F11), PE-conjugated Siglec F (clone E50-2440), PECy7-conjugated CD11c (clone HL3), APC/Cy7-conjugated CD4 (clone GK1.5), PerCP/Cy5.5-conjugated Gr-1(clone RB6-8C5) and APC-conjugated CD11b clone (M1/70). The antibodies were purchased from Biolegend (San Diego, U.S.), BD Biosciences, eBioscience (Hatfield, UK) or MD Bioproducts (Zürich, Switzerland).

### Statistical analysis

Statistical analyses were performed using Graph pad Prism, version 6.05 (GraphPad Software, San Diego, CA, USA). Results are expressed as mean ± SEM. ANOVA followed by Bonferroni’s post correction were used to compare differences between groups. Chi-square tests for trends were used to analyze semi-quantitative scoring data. A P-value of <0.05 was considered statistically significant.

## Electronic supplementary material

Interleukin 33 exacerbates antigen driven airway hyperresponsiveness, inflammation and remodeling in a mouse model of asthma
