# Supplementary material for: Interleukin 33 exacerbates antigen driven airway hyperresponsiveness, inflammation and remodeling in a mouse model of asthma
Source: Sci Rep. 2017 Jun 26;7:4219. doi: 10.1038/s41598-017-03674-0 (PMC5484710; doi:10.1038/s41598-017-03674-0)
Supplement: Supplementary file 1 — Interleukin 33 exacerbates antigen driven airway hyperresponsiveness, inflammation and remodeling in a mouse model of asthma [file 41598_2017_3674_MOESM1_ESM.pdf]

Supplementary Information

Title: Interleukin 33 exacerbates antigen driven airway hyperresponsiveness, inflammation and remodeling in a mouse model of asthma

Authors: LC Sjöberg[1,*], A Zoltowska Nilsson[2,*], Y Lei[2], JA Gregory[1, 3], M Adner[1, 3, #] and GP Nilsson[2, 3, #]

Affiliations:

[1] Unit of Experimental Asthma and Allergy Research, Institute of Environmental Medicine; [2] Immunology and Allergy Unit, Department of Medicine; [3] Centre for Allergy Research, Karolinska Institutet, and Karolinska University Hospital, Stockholm, Sweden

[*] These authors contributed equally to this study.

[#] These authors share senior authorship

Corresponding author:

Gunnar Nilsson, Department of Medicine, Solna, Immunology and Allergy Unit, Karolinska Institutet, KS L2:04 171 76 Stockholm

E-mail: Gunnar.P.Nilsson@ki.se; Phone: +46 8 517 702 05

a

b

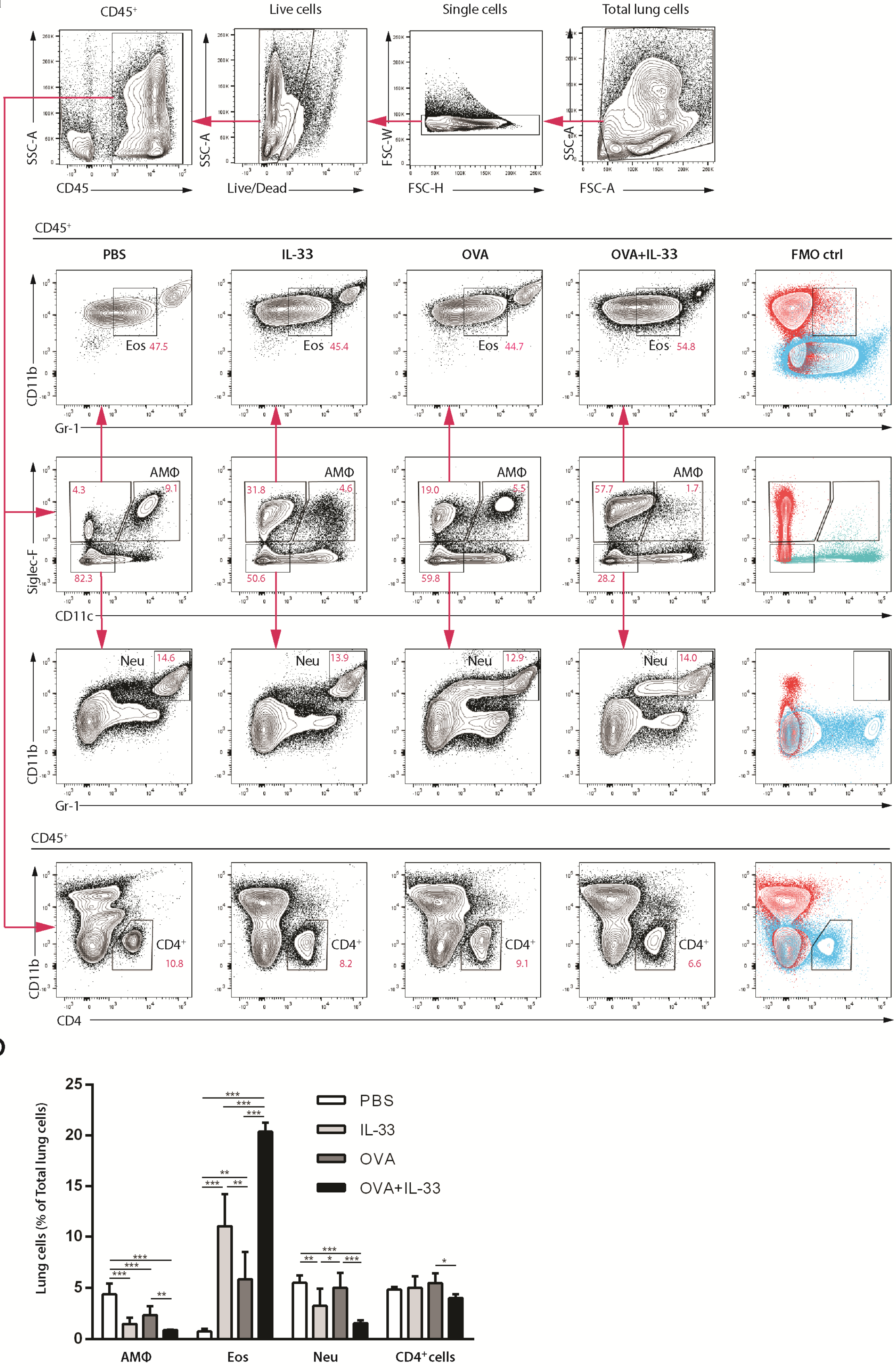
CD45+
Live cells
Single cells
Total lung cells
SSC-A
CD45
Live/Dead
FSC-W
FSC-H
FSC-A
PBS
IL-33
OVA
OVA+IL-33
FMO ctrl
Eos 47.5
Eos 45.4
Eos 44.7
Eos 54.8
CD11b
Gr-1
AMΦ
4.3
9.1
82.3
31.8
4.6
50.6
19.0
5.5
59.8
57.7
1.7
28.2
Siglec-F
CD11c
Neu 14.6
Neu 13.9
Neu 12.9
Neu 14.0
CD4+ 10.8
CD4+ 8.2
CD4+ 9.1
CD4+ 6.6
CD4
Lung cells (% of Total lung cells)
0
5
10
15
20
25
AMΦ
Eos
Neu
CD4+ cells
PBS
IL-33
OVA
OVA+IL-33

Supplementary Figure S1. Flow cytometric analysis of inflammatory lung cells. (a) Gating strategy and representative plots for each exposure group. Alveolar macrophages were identified as $CD45^{+}$ $SiglecF^{+}$ $CD11c^{+}$, eosinophils as $CD45^{+}SiglecF^{+}CD11c^{-}CD11b^{+}Gr\text{-}1^{lo}$, neutrophils as $CD45^{+}$ $SiglecF^{-}$ $CD11c^{-}Gr\text{-}1^{hi}CD11b^{hi}$ and $CD4^{+}$ cells as $CD45^{+}CD11b^{-}$ $CD4^{+}$. Gates were set based on FMO controls, FMO ctrl= overlay of the two FMO controls for each respective plot. The representative FMO plots and plots showing the representative $SSC^{low}FSC^{low}CD45^{+}$ gating belong to the OVA+IL-33 group. (b) Lung frequencies of alveolar macrophages, eosinophils, neutrophils and $CD4^{+}$ cells. *$p<0.05$, **$p<0.01$, ***$p<0.001$ (ANOVA, Bonferroni). Results are pooled data of four independent experiments (mean ±SEM of n=8 mice in each group).
